# Measuring the Enterprise Green Innovation Strategy Decision-Making Quality: A Moderating—Mediating Model

**DOI:** 10.3389/fpsyg.2022.915624

**Published:** 2022-06-13

**Authors:** Jiaying Feng, Ying Pan, Wencan Zhuang

**Affiliations:** ^1^School of Economics and Management, Harbin University, Harbin, China; ^2^School of Economics and Management, Harbin University of Science and Technology, Harbin, China

**Keywords:** green decision, board capital, group Faultline, green innovation, SEM

## Abstract

Green development helps to balance the conflict between economic expansion, environmental protection, and green strategy decisions by tackling the issue of excessive resource utilization during regional growth. This study aims to measure the green innovation strategic decisions quality by identifying the nexus between board capital, green innovation strategic information acquisition capability, and board group Faultline. A conceptual model has been proposed and tested to verify the proposed relationship. Data collection was analyzed using structural equation modeling in AMOS 24.0. The findings indicate that board human capital (BHC) and board social capital (BSC) have a beneficial influence on the quality of green innovation strategy decision-making. The green innovation strategic information acquisition capability plays a mediating role in the relationship between the two dimensions of board capital and the green innovation strategy decision-making quality. The mediating role of green innovation strategic information acquisition capability is moderated by board group Faultline. The core significance of this study is presented.

## Introduction

With the increasingly prominent environmental and resource issues, green technology innovation is a new trend of technological innovation (Schiederig et al., 2012). Under the concept of sustainable development, with resource conservation and environmental protection as the core, and the pursuit of sound and rapid development as the starting point and endpoint. The organizational green innovation strategy is the overall goal deployment of innovation activities, and the overall plan and fundamental countermeasures to achieve the goal of green innovation (Song and Yu, 2018). Whether an enterprise can obtain and maintain a competitive advantage by cultivating green innovation depends on whether the green innovation strategic decision is correct or not (Zhang et al., 2020a). Therefore, to improve the quality of green innovation strategic decision-making urgently needs theoretical guidance. In the main context of enterprise technology innovation strategy decision-making, the board of directors participates in the whole process of formulation, implementation, control, and evaluation of green innovation strategy (Rui-Zhi et al., 2019; Valenti and Horner, 2020). Board capital is a combination of various professional knowledge and vocational skills possessed by board members and the social network of the board (Wincent et al., 2010). As the basis of the board's participation in innovation strategy, the relationship between board capital and the innovation strategy decision-making quality (DQ) is a hot issue in the research field of innovation strategy (Sarto et al., 2019).

Many scholars put forward that board capital has a positive influence on the innovation strategy DQ through theoretical analysis and empirical evidence. Literature pointed out that board capital can provide enterprises with various resources, including suggestions and consulting complementary technology, internal and external environmental information, and financial resources, which is conducive to the improvement of innovation strategic DQ (Liu et al., 2010; Sarto et al., 2019; Kontesa et al., 2020; Shahzad et al., 2022). The paper by Wang (2021) explores the impact of BHC diversity on firm innovation and finds a positive moderating effect of BSC on firm innovation. However, some scholars hold the opposite view. For example Azevedo (2021), found that when levels of board capital are not very different, different enterprises have made innovative strategic decisions with different quality. Lin et al. (2006) conducted an empirical study on the relationship between board capital and the innovation strategy DQ, and found that the impact of board capital on innovation strategy DQ is not significant. Some scholars even believe that the relationship resources of the board may be negatively related to the innovation strategy DQ, because the relationship is a “double-edged sword,” which brings resources and may also cause the organization to rely on other roles (Dakhli and De Clercq, 2004).

The influence of board capital on the quality of innovation strategy decision-making is the function of the board of directors to provide resources in the process of innovation strategy decision-making (Barney, 2001). The direction of influence was directly discussed, but no consensus was reached. The decision-making process of innovation is essentially a process of information flow. The board of directors is required to collect and sort out the information in each link of the strategic decision-making of innovation to support the effective decision-making process (Wincent et al., 2010). Different boards of directors have different levels of capital, which means that there are differences in overall skills, experience, and social relations of the board of directors, as well as the ability to obtain strategic information of innovations.

As an open team at the institutional level, it is difficult for the board of directors to achieve complete standardization through formal rules and procedures to promote the exertion of capital. In the process of obtaining green innovative strategic information, the effective exertion of board capital depends on the behavior intention of board members to a great extent (Wang, 2021). The behavior willingness of board members is essentially a kind of social psychological process, so the problems related to the social dynamics within the board, that is, the group Faultline, need to be considered (Bezrukova et al., 2012). The group Faultline formed by the multiple attribute characteristics of the members within the board will make the board have an internal sharing equilibrium, which will affect the degree of effort of the board members, and then affect the board members (Thatcher and Patel, 2012). Therefore, this study analyzes the extent to which board capital plays a role in the process of green innovation strategic information acquisition. Meanwhile, the influence of board capital on the DQ of green innovation strategy is discussed in the literature. Furthermore, the mediating effect of the information acquisition ability of green innovation strategy and the influence of board capital on the DQ of green innovation strategy were also analyzed (Shin and You, 2019; Ali and Ayoko, 2020). In the context of social norms and based on the faultline theory, the impact of board faults on innovation strategic decisions of listed companies in technology-intensive industries in China from 2009 to 2015 was examined (Zhang and Ma, 2022). Therefore, this study has been performed to answer the following research questions.

1) What is the impact of board capital on green innovation strategic information acquisition capacity (IAC) leading to the green innovation strategy DQ?
2) How BGF moderates the relationship between board capital and green innovation strategic IAC?

The findings of this study can not only deepen the understanding of the relationship between the board capital and the quality of green innovation strategic decision-making but also provide a valuable orientation for the board to use its funds to improve the quality of green innovation strategic decision-making management practices.

## Literature Review and Research Hypotheses

### Cognitive-Behavioral Theory

Early theories of substance abuse behavior were disintermediated. They focus almost exclusively on overt, observable behavior, and it is believed that understanding antecedents and reinforcing contingencies is sufficient to explain and modify behavior (Wilson, 1999). Over time, however, these behavioral theories began to incorporate cognitive factors into their conceptualizations of substance-use disorders (Gagné and Deci, 2005; Benson et al., 2019). The cognitive-behavioral theory is an integration of behavioral and cognitive theory that provides a more inclusive and comprehensive approach to the treatment of substance-use disorders. However, this theory covers a wider range of cognition than earlier versions of cognitive theory. This theory also points out that information acquisition is a process of thinking emotions and behavioral interaction of individuals, and individual differences such as knowledge reserve, experience, and social network relationship of information collectors all affect information acquisition ability (Wilson, 1999). Several studies in the literature employ cognitive-behavioral theory from different perspectives to measure the cognitive behavior of individuals and is considered a key aspect of organizational decision-making, i.e., in entrepreneurship (Shahzad et al., 2021), technology adoption decisions (Park and Kim, 2014; Malik et al., 2016; Zhang, 2019), social media and consumer behavior (Wang and Lin, 2012; Wang et al., 2019). Therefore, considering the importance of this theoretical aspect, we use it as the underlying theory for determining the strategic decision-making behavior of an organization's green innovation.

### Relationship Between Board Capital and the Green Innovation Strategy Decision-Making Quality

Hillman and Dalziel (2003) created the notion of board capital in the field of corporate governance. They proposed that board capital is the capability of the board to offer available resources for enterprises. As a result, boards with greater age diversity have a more active oversight role in voting for the use of green innovation to develop green products (Xia et al., 2022). Later, Wincent et al. (2010) defined the connotation of board capital from the perspective of its existing form. They believed that board capital is the general term of experience, skills, and social network relations possessed by board members, and divided board capital into two dimensions: human capital and social capital. Board human capital (BHC) is the general term for intangible assets such as knowledge, skills, experience, and reputation possessed by board members. Board social capital (BSC) refers to the collection of connections between all members of the board and other members of the external organization. This study is conducted from the two dimensions of BHC and BSC.

Enterprise green innovation is the sum of a series of technological innovations and management innovations that pursue sound and rapid development of enterprises under the guidance of sustainable development values, with resource conservation and environmental protection as the core (Zhang et al., 2020b). Other studies have defined green innovation as a method of producing innovative and competitive goods, facilities, processes, procedures, and edifices that use natural resources wisely and promote healthy lifestyles (Karimi Takalo et al., 2021; Hu et al., 2022). It takes technology, economy, and ecology as the elements of mutual promotion, and is a comprehensive innovation activity integrating ecology, science, and institutionalization. Methods, timing, and thumbprint of resources invested by enterprises in green innovation constitute the content of strategic decisions for technology innovation (Schiederig et al., 2012). Scholars at home and abroad interpret the connotation of innovation strategy DQ mainly based on six criteria of strategic DQ evaluation proposed by Haas et al. (1981). As per the said criteria, it is considered whether the strategic decision-making is consistent with the enterprise's objectives; whether the strategic decision-making is suitable for the key resources inside the enterprise; whether the strategic decision-making is consistent with the external environment of the enterprise; whether the risk of strategic decision-making is within the scope that the enterprise can bear; whether the strategic decision-making determines the appropriate time to achieve the goal; and whether the strategic decision-making achieves the expected effect. In this study, integrating the existing research literature on the green innovation strategy decision process, the green innovation strategy DQ is defined as the degree to which the green innovation strategy is consistent with the external environment, internal resources, and capabilities of the enterprise as well as the goal of green innovation.

Several valuable resources related to the survival and development of an organization need to be obtained from the external environment. The board is an important contact mechanism between the enterprise and the environment, which can help the enterprise to obtain resources (Ewer et al., 2019). According to Johnson et al. (1996), the more abundant BHC, the higher level of technical ability, knowledge, and experience of the board, which can promote the legitimacy and reputation of the enterprise. A higher corporate reputation can improve the recognition of external investors for technology innovation behavior and help the enterprise obtain financial resources (De Maere et al., 2014). The relationship between the board and the stakeholders in the supply chain, such as manufacturers, customers, suppliers, distributors, strategic partners, etc., is conducive to the effective communication between the enterprise and its partners, the rapid establishment of a common language, and the promotion of the enterprise's access to external information, technology, knowledge, and other resources (Chen, 2014).

Furthermore, a high-quality strategic decision of green innovation requires that it can match the external environment and internal resource capacity, but most enterprises are often constrained by internal resources and cannot make a green innovation strategy to adapt to the external environment (Nutt, 2008). The higher the board of directors' level of human capital and social capital, the more abundant resources are provided for the enterprise, the fewer resource constraints are imposed on the enterprise's green innovation strategic decision-making, and the green innovation strategic decision-making plan is formed, resulting in improved green innovation (Lin et al., 2006; Makaryanawati, 2019; Ramón-Llorens et al., 2019). Based on the above, this study proposes the following assumptions:

**H1a:** BHC is positively related to the green innovation strategy DQ.
**H1b:** BSC is positively related to the green innovation strategy DQ.

### Relationship Between Board Capital and the Ability to Acquire Strategic Information on Green Innovation

The concept of technology innovation strategic IAC originated from the field of library and information. Information scientists Nahl and Tenopir (1996) defined IAC as “the capacity to use information resources in work after training.” With the increasing importance of information in strategic decision-making, the concept of IAC has gradually penetrated the strategic field. The study of Wakolbinger and Cruz (2011) states that the capacity to acquire strategic information refers to the ability to acquire, query, exchange, disseminate, absorb, and process information under the stimulation of external information sources to meet the information needs of strategic decision-making.

Based on the theory of cognitive behavior, individual differences such as the interaction between individuals and the environment and the experience accumulated by individuals in the course of life can affect cognitive ability (Wilson, 1997). The cognition and expression stage of information needs the selection stage of information sources, and the absorption stage of information are all cognitive related activities, and cognitive ability plays a unique and important role in the process of information acquisition (O'Brien and Symons, 2005). Different boards have different levels of capital, which means that their overall skills, experience, and social relationships will vary, as will their cognitive abilities, as well as their ability to access information about green innovation strategies (Valenti and Horner, 2020; Azevedo, 2021).

The process of potential information demand transformation is a deconstruction of an individual's original cognitive model and the construction of a new cognitive model. Learning factors are “catalysts” in the cognitive process of transformation of potential information demand (Rafferty and Gary, 2016). If BHC is high, it means that the overall memory capacity of the board is high (Ramón-Llorens et al., 2019), and it is easy to accept new necessary knowledge, then the board will quickly convert the potential strategy decision-making information demand of green innovation into the actual strategy decision-making information demand of green innovation. The process of “behavioral interaction,” is accompanied by the “cognitive interaction” of the individual's inner world, that is, conscious thinking and information in the sense of informatics (Ramón-Llorens et al., 2019). From the perspective of information processing, cognitive psychology proposes that the rational knowledge of information users directly affects the results of cognitive interaction. After clearly expressing the demand for green innovation strategy information, the board of directors needs to choose the path to obtain green innovation strategy information, and then conduct “behavioral interaction” with the information source to obtain the information provided by the information source (Kontesa et al., 2020). The higher the BHC, the richer the rational knowledge of the board members as a whole, the stronger their thinking ability (Makaryanawati, 2019), and the better the cognitive interaction effect with the information source (Kontesa et al., 2020). The board of directors can make a comprehensive, reasonable, and objective judgment on each feedback information of the information source, and accordingly construct the next information behavior strategy purposefully and step by step.

The cognitive structure of a cognitive object fundamentally determines the direction of understanding the object information (González-Valiente et al., 2019). From this point of view, cognitive structure plays a decisive role in the absorption and transformation of information. Individuals cultivate their knowledge and skills through interaction with others in the environment and enrich their cognitive structure (Wood and Bandura, 1989). The daily social interaction activities and processes of the board are the processes of interaction with the environment, which is a process of cognition and learning. The study of Dakhli and De Clercq (2004) pointed out that the higher the BSC, the more frequent and close the communication between board members and social network members. The more opportunities for information and knowledge sharing, the deeper the level of communication, which helps to improve the acquisition, horizontal integration, and utilization of knowledge, especially intangible knowledge (Wang, 2021). Therefore, the higher BSC, the more complex the understanding structure of the board members as a whole, the more information they can receive from the object, the more comprehensive the knowledge they form, and the stronger the ability to respond appropriately to the stimulation of the object information and to process it. Based on the above analysis, this study proposes the following assumptions:

**H2a:** BHC is positively related to the board's green innovation strategic IAC.
**H2b:** BSC is positively related to the board's green innovation strategic IAC.

### Relationship Between the Green Innovation Strategic Information Acquisition Capacity and the Green Innovation Strategy Decision-Making Quality

The studies point out that market information, technology information, policy information, and other technology innovation strategic information, as an intangible resource, is very important to the process of innovation strategy decision (Barney, 2001). However, the strategic information of green innovation is not a continuous variable, which has the characteristics of complexity and dynamic (Li et al., 2019). There is no historical model or outline to be referenced, and it is typical blind information. The ability of the board of directors to obtain green innovation strategic information determines the level of green innovation strategic information, which in turn affects the quality of green innovation strategic decision-making (Xia et al., 2022). The influence of green innovation strategic IAC of the board on the green innovation strategy DQ is mainly reflected in the following three aspects: first, the board's strong acquisition ability of green innovation strategic information can improve the accuracy of the innovation strategy environment assessment. According to the assumption of limited rationality of humans, decision-makers cannot have insight into all aspects of the internal and external environment of the organization, which is likely to cause the limitations of decision-makers' understanding (Forbes, 2007).

The strong green innovation strategic IAC can obtain perfect strategic decision-making information on technology innovation, enrich the knowledge structure of directors, reduce the cognitive deviation of the board on the internal and external situation of the enterprise, and improve the accuracy of the assessment of the strategic environment of green innovation (Meissner and Wulf, 2013). Second, the strong green innovation strategic IAC can improve the flexibility of the green innovation strategy (Müller et al., 2021). The environment of green innovation strategy is highly uncertain and dynamic. The stronger green innovation strategic IAC, the faster access to the strategic information of green innovation. On this basis, more alternative schemes of green innovation strategy are designed to improve the flexibility of green innovation strategy. Third, the strong green innovation strategic IAC can improve the ability of decision-making and evaluation of green innovation strategy. The stronger the board has access to technology innovation strategic information, the more abundant the board of directors has access to green innovation strategic decision-making information. A comprehensive and objective evaluation of the strategic plan can ensure that a higher-quality strategic plan can be selected from several strategic decision-making plans for green innovation. Based on the above analysis, the following assumptions are proposed:

**H3:** Green innovation strategic IAC of the board is positively related to the green innovation strategy DQ.

### Median Role of the Green Innovation Strategic Information Acquisition Capacity

BHC determines the overall memory capacity and rational knowledge depth of the board. BSC enriches the cognitive structure of the board (Wang, 2021). Both of them promote the acquisition ability of the strategic information of the board of directors, which makes them have more accurate, perfect, and timely strategic information when making strategic decisions, and then make a high-quality strategic decision of green innovation. It can be concluded that the impact of board capital on the green innovation strategy DQ is to a certain extent realized through the influence on the green innovation strategic IAC, that is, the green innovation strategic IAC plays a median role between the board capital and the technology innovation strategy DQ. Based on the above analysis, this paper proposes the following assumptions:

**H4a:** The green innovation strategic IAC plays a mediating role in the relationship between BHC and the green innovation strategy DQ.
**H4b:** The green innovation strategic IAC plays a mediating role in the relationship between BSC and the green innovation strategy DQ.

### Moderating Effect of the BGF

The improvement of the BHC and BC provides the possibility for the improvement of green innovation strategic IAC. However, the degree of improvement depends on the degree to which the BHC and the BSC play a role (Azevedo, 2021). A group fault line is a group differentiation formed by the combination of different characteristics of team members (Zhang and Ma, 2022). The BGF is a set of hypothetical segmentation lines that divides the board of directors into several subgroups based on the multiple attribute characteristics of the board members (Crucke and Knockaert, 2016). As a prospective manifestation of group division, the existence of Faultline makes the board of directors split into several similar subgroups. The role of social classification and social identity will deepen the hostility and vicious competition among members of different subgroups, affect the efforts of board members, and then affect the effective use of board capital (Ramón-Llorens et al., 2019; Valenti and Horner, 2020).

The information demand of innovation strategy realized by a single director may be limited to a particular field, lacking certain universality and systematic comprehensiveness. The directors are familiar with each other and exchange information needs through cognition (Hillman and Dalziel, 2003). In the process of information demand sharing of strategic decision-making, psychological security is very important. In the environment of a weak Faultline, group members may be more willing to express their opinions without considering the possibility of being rejected, to improve the willingness to share information needs of innovation strategy (Thatcher et al., 2003). In addition, due to the multiplicity of strategic decision-making objectives of green innovation, the uncertainty of the environment, and the dynamics of time, the green innovation strategic IAC often goes beyond the scope of individual ability, which requires the cooperation between board members. In the environment of a strong Faultline, the perception of “within group—outside group” strengthens the boundary among different subgroups, and members will give a positive evaluation of their subgroups, respectively. Meanwhile, the negative evaluation will be imposed on other groups, so that the recognition of board members to the subgroups is stronger, even more than the recognition of the board as a decision-making whole. This will weaken the internal cohesion of the board and reduce their willingness to cooperate in innovation strategy decision-making (Lim et al., 2013). Therefore, it can be considered that the degree of BGF harms the extent of the board capital in the process of obtaining innovative strategic information.

Based on the above analysis, it can be concluded that the BGF negatively regulates the impact of board capital on the ability to obtain green innovation strategic information. The ability to obtain green innovation strategic information plays a median role in the relationship between board capital and green innovation strategy DQ. Therefore, the BGF will have a negative regulatory effect on the intermediary role of the ability to obtain innovation strategic information in the relationship between board capital and the quality of innovation strategic decision-making. The board capital has a great influence on the ability to obtain innovative strategic information (Shin and You, 2019; Ali and Ayoko, 2020). The ability to obtain innovation strategic information more transmits the effect of the green innovation strategy DQ. On the contrary, when the BGF is strong, even if the board capital is high, it cannot play an effective role. The green innovation strategic IAC is also weak, and the ability to obtain innovation strategic information plays less role in the green innovation strategy DQ. Based on the above analysis, the following assumptions are proposed:

**H5a:** The BGF regulates the moderating role of the green innovation strategic IAC in the relationship between BHC and the technology innovation strategy DQ.
**H5b:** The BGF regulates the moderating role of the green innovation strategic IAC in the relationship between BSC and the green innovation strategy DQ.

**Figure 1:**
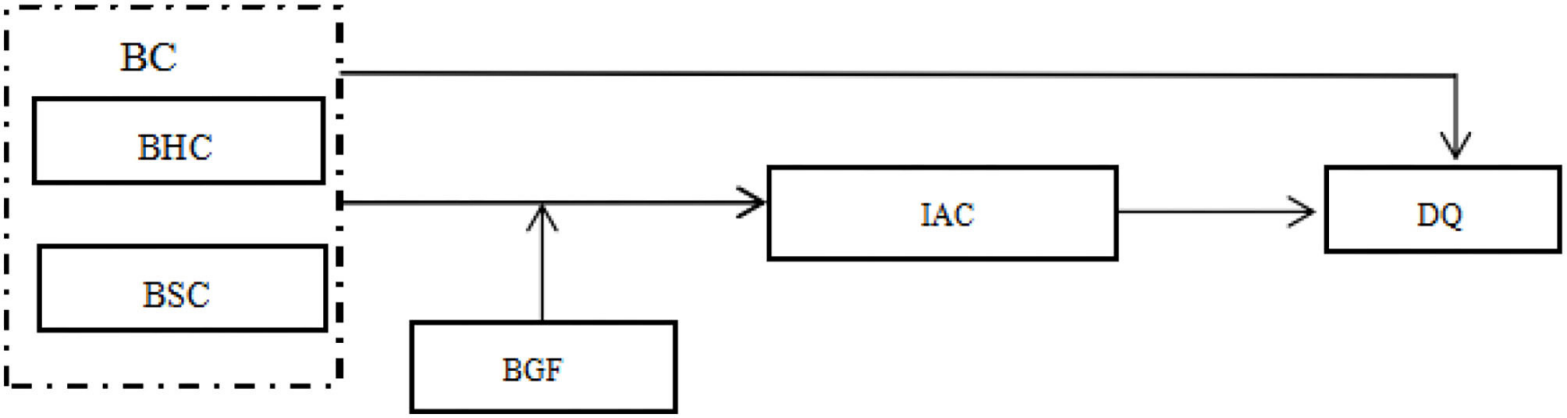
The conceptual model.

Based on the above research assumptions, the conceptual model is shown in Figure 1.

## Research Methodology

### Variable Measurement

The measurement of BHC mainly refers to the research by Fischer and Pollock (2004) and Dakhli and De Clercq (2004), and selects three indicators to reflect the education level of the board, the term of office of the board, and the professional background of the board. Among them, the education level of the board is measured by the average value of the highest education level of each board member (the highest education level of the board is assigned as 4, 3, 2, and 1 according to the doctorate graduate student, master graduate student, undergraduate student, and below undergraduate student, respectively). The tenure of the board is measured by the average value of the number of years that the board members have served as directors in the enterprise, and the functional background of the board is measured by the proportion of the number of directors with marketing, product design, and R&D functional background.

The measurement of BSC mainly refers to the research of Wincent et al. (2010) and Jermias and Gani (2014), which select three indicators to reflect the corporate relationship, political relationship, and financial relationship. Among them, the corporate relationship is measured by the proportion of chain directors in the total number of the board members. The financial relationship is measured by the proportion of directors who are or have served in financial institutions in the total number of the board, and the political relationship is measured by the proportion of directors who are or have served in state organs in the total number of the board. The measurement of BGF is mainly based on research (Bezrukova et al., 2007; Trezzini, 2008), and three indicators are selected to reflect the strength, width, and depth of the BGF. Among them, the strength of the BGF is quantified by the Faun algorithm, the width of the BGF is measured by the degree of dispersion between subgroups, and the depth of the BGF is measured by the Euclidean distance between the subgroups' centroids.

The measurement of the green innovation strategic IAC is mainly based on the work of Thomas et al. (2009), and four items related to the timeliness and cost of information acquisition are selected, such as “the board of directors can obtain the required green innovation strategic information in time,” etc. The measurement of the green innovation strategy DQ is mainly based on (Dooley and Fryxell, 1999) the strategic decision quality scale. It reflects the quality of green innovation strategy decision-making from the aspects of environmental consistency, internal consistency, adaptability, risk degree, and effectiveness of green innovation strategy. There are six items in total, such as “green innovation strategy is consistent with the market environment,” etc. Likert's five-level scale was used to measure the ability to obtain information and decision-making on green innovation strategy, with 1 indicating “very disagree” and 5 indicating “very agree.” The measurement indexes and items of each variable are shown in Table 1.

**Table 1:** Variables indicators and items.

**Variable's name**	**Code**	**Indicators and Measures**
Board Human capital (BHC)	BHC_1_	Director's education level
	BHC_2_	Duration of the Board of Trustees
	BHC_3_	Board functional background
Board Social Capital (BSC)	BSC_1_	Board corporate relations
	BSC_2_	Board financial relations
	BSC_3_	Board political relations
Board Group Faultline (BGF)	BGF_1_	Strength of board group Faultline
	BGF_2_	Width of board group Faultline
	BGF_3_	Depth of board group Faultline
Innovation strategic information acquisition capacity (IAC)	IAC_1_	Boards have extensive access to green innovation strategy information
	IAC_2_	Timely access by the board to the required green innovation strategy information
	IAC_3_	The board has access to a large number of green innovative strategy information
	IAC_4_	Board access to green innovation strategy information at a lower cost
Innovation strategy decision-making quality (DQ)	DQ_1_	Innovation strategy is aligned with the market environment
	DQ_2_	Innovation strategy and policy environment are aligned
	DQ_3_	The innovation strategy reflects the company's current financial position
	DQ_4_	Innovation strategy is aligned with internal resources and capabilities
	DQ_5_	Innovation strategy is adapted to other company decisions
	DQ_6_	Innovation strategy is compatible with the organizational structure of the enterprise
	DQ_7_	Innovation strategy promotes the achievement of innovation goals

### Sample Selection and Data Collection

Take the enterprises with green innovation as the research samples. Data of board capital and BGF are from the resume of the board disclosed in the company's annual report. The data of the green innovation strategy DQ and the green innovation strategic IAC are obtained using a questionnaire survey, in which the quality scale of green innovation strategic decision-making is assessed by the executive in charge of the implementation of green innovation strategic decision-making, and the ability scale of obtaining green innovation strategy information is assessed by the chairman of the board.

Obtained the BHC and BSC data of 350 enterprises. Among them, 230 enterprises surveyed the paper form, and 120 enterprises surveyed the electronic form. Two questionnaires were sent out to each enterprise, 700 questionnaires were distributed, and 535 questionnaires were recovered. After eliminating the incomplete questionnaires and those filled out only by the chairman or senior managers, 462 valid questionnaires were finally obtained from 231 enterprises, with an effective response rate of 66%.

### Data Standardization

Among the variable data obtained, there are not only the subjective data of level 1–5 scale of the green innovation strategic IAC and the green innovation strategy DQ but also the objective data of board capital and BGF. Due to the differences in properties and dimensions, the representation methods of each variable are also different, including quantity value, grade, percentage, etc., resulting in the incompatibility and consistency between variables. Therefore, it is necessary to standardize variable data. This paper uses the hierarchical membership function method to convert the objective actual monitoring index data into 1–5 grade values so that the processed indexes and grade items are consistent. First of all, according to formula (1), calculate the mean value *d*^*i*^ of interval distance between BHC and BSC, where *a*^*i*^ is the maximum value of the index *i* and *b*^*i*^ is the minimum value of index *i*. Second, according to formula (2), calculate the grade interval boundary value ${k_{n}}^{i}$ of index*i*, where *n* is the grade number and the value is 1, 2, …, 5. Finally, the actual value of index *i* is determined to belong to the grade interval, and the corresponding grade value is taken.


(1) $$dj=\left|\frac{\left|ai\right|-\left|bi\right|}{4}\right|$$

(2) $$\begin{array}{r} k_{n}^{i}=k_{1}^{i}+\left(n-1\right)d \end{array}$$

## Results and Discussion

### Reliability and Validity Analysis of Variables

The reliability of all variables in this paper is tested by SPSS 21.0. By using the method of the structural equation model and AMOS 24.0, a confirmatory factor analysis of the theoretical measurement model including all variables in this study is carried out to test the convergence validity, discrimination validity, and combination reliability (CR) of variables. The inspection results are shown in Table 2.

**Table 2:** Reliability and validity of variables.

**Variables**	**Term**	**Loadings**	**Cronbach's α**	**AVE**	**CR**
BHC	BHC_1_	0.772	0.788	0.608	0.822
	BHC_2_	0.769			
	BHC_3_	0.797			
BSC	BSC_1_	0.788	0.834	0.625	0.834
	BSC_2_	0.834			
	BSC_3_	0.748			
BGF	BGF_1_	0.843	0.893	0.683	0.866
	BGF_2_	0.779			
	BGF_3_	0.855			
IAC	IAC_1_	0.759	0.864	0.615	0.864
	IAC_2_	0.803			
	IAC_3_	0.785			
	IAC_4_	0.788			
DQ	DQ_1_	0.802	0.927	0.646	0.927
	DQ_2_	0.866			
	DQ_3_	0.814			
	DQ_4_	0.788			
	DQ_5_	0.862			
	DQ_6_	0.781			
	DQ_7_	0.798			

*BHC, Board Human capital; BSC, Board Social Capital; BGF, Board Group Faultline; IAC, Innovation strategic information acquisition capacity; DQ, Innovation strategy decision-making quality*.

The results of reliability analysis show that Cronbach's α value of each variable is greater than the standard of 0.70, indicating that the latent variable has good reliability (Myon et al., 2005). The confirmatory factor analysis of the measurement model shows that c2/df = 1.031, <2.0; RMSE = 0.012, <0.05; GFI = 0.924, CFI = 0.998, TLI = 0.997, all of which exceeded the specified critical value of 0.90, and the overall fitness index of the measurement model met the requirements (Byrd and Turner, 2000). It shows that the measurement model and sample data fit well. Further factor analysis shows that the factor loads of all measures are >0.70, indicating that the variables have good convergence validity (Baron and Kenny, 1986). The CR of each variable is more than the critical value of 0.70 (Yang et al., 2010), indicating that the variable has good CR. The mean-variance extraction value (AVE) of each variable was >0.50 (Thong et al., 1996), which indicated that the variable had good discrimination validity. In general, the variable measurement model has a good validity structure, which can be further analyzed.

### Descriptive Statistics and Correlation Analysis

To test the mechanism of the board capital on the green innovation strategy DQ, it is necessary to first determine the correlation between variables. SPSS 21.0 is used to analyze the correlation of each variable. During the correlation analysis, the mean value of all items (indicators) of a variable is taken as the value of the variable. It can be seen from Table 3 that BHC, BSC, the green innovation strategy DQ, and the green innovation strategic IAC are significantly positively correlated, which can be further analyzed.

**Table 3:** Means, standard deviations, and correlation coefficients among variables.

**Variables**	**Mean**	**St. D**	**BHC**	**BSC**	**BGF**	**IAC**	**DQ**
BHC	2.33	1.17	1				
BSC	2.29	1.21	0.064*	1			
BGF	2.17	1.07	0.117**	0.162**	1		
IAC	3.30	1.14	0.535***	0.487***	0.455**	1	
DQ	3.17	1.18	0.570**	0.521**	0.724***	0.196**	1

***p < 0.001, **p < 0.005, *p < 0.01.

### Test of Main Effect and Median Effect

To test hypotheses 1a−4b, five nested structural equation models are constructed by structural equation model analysis. M1 is a partial intermediary model, including the direct and indirect influence of the two dimensions of board capital on the green innovation strategy DQ; M2 is a complete intermediary model, including the indirect influence of the two dimensions of board capital on the green innovation strategy DQ; M3 is a non-intermediary model, which includes the direct influence of the two dimensions of board capital on the green innovation strategic IAC and the direct influence of the two dimensions of board capital on the green innovation strategy DQ; M4 is a non-intermediary model, which includes the direct impact of the two dimensions of board capital on the green innovation strategy DQ, and the direct impact of the green innovation strategic IAC on the green innovation strategy DQ; M5 is a non-intermediary model, which includes the two dimensions of board capital have a Positive impact on the green innovation strategy DQ. According to the fitting index of each structural equation model, the optimal model is selected, and then the above hypothesis is tested according to the path coefficient of the optimal model. In this study, AMOS 24.0 is used to detect the structural model, and the fitting results of five structural equations are shown in Table 4.

**Table 4:** Nested comparison results of SEM.

**Team**	**M1 Partial mediating role**	**M2 Totally mediating**	**M3 No mediating**	**M4 No mediating**	**M5 No mediating**
Standardized path coefficient				
BHC-DQ	0.34***	–	0.59**	0.38***	0.54**
BSC-DQ	0.31***	–	0.52**	0.35***	0.49**
BHC-IAC	0.51***	0.54***	0.57**	–	–
BSC-IAC	0.45***	0.19***	0.49**	–	–
GIAC-DQ	0.39***	0.76**	–	0.51**	–
Model fit index				
Df	113	115	114	115	116
χ^2^	133.63	134.15	132.03	138.25	139.43
GFI	0.938	0.926	0.929	0.906	0.894
TLI	0.989	0.975	0.980	0.938	0.915
CFI	0.991	0.979	0.983	0.948	0.928
RMSEA	0.028	0.043	0.038	0.067	0.078
χ^2^/df	0.846	0.857	0.863	0.832	0.832

***p < 0.001, **p < 0.005.

**Figure 2:**
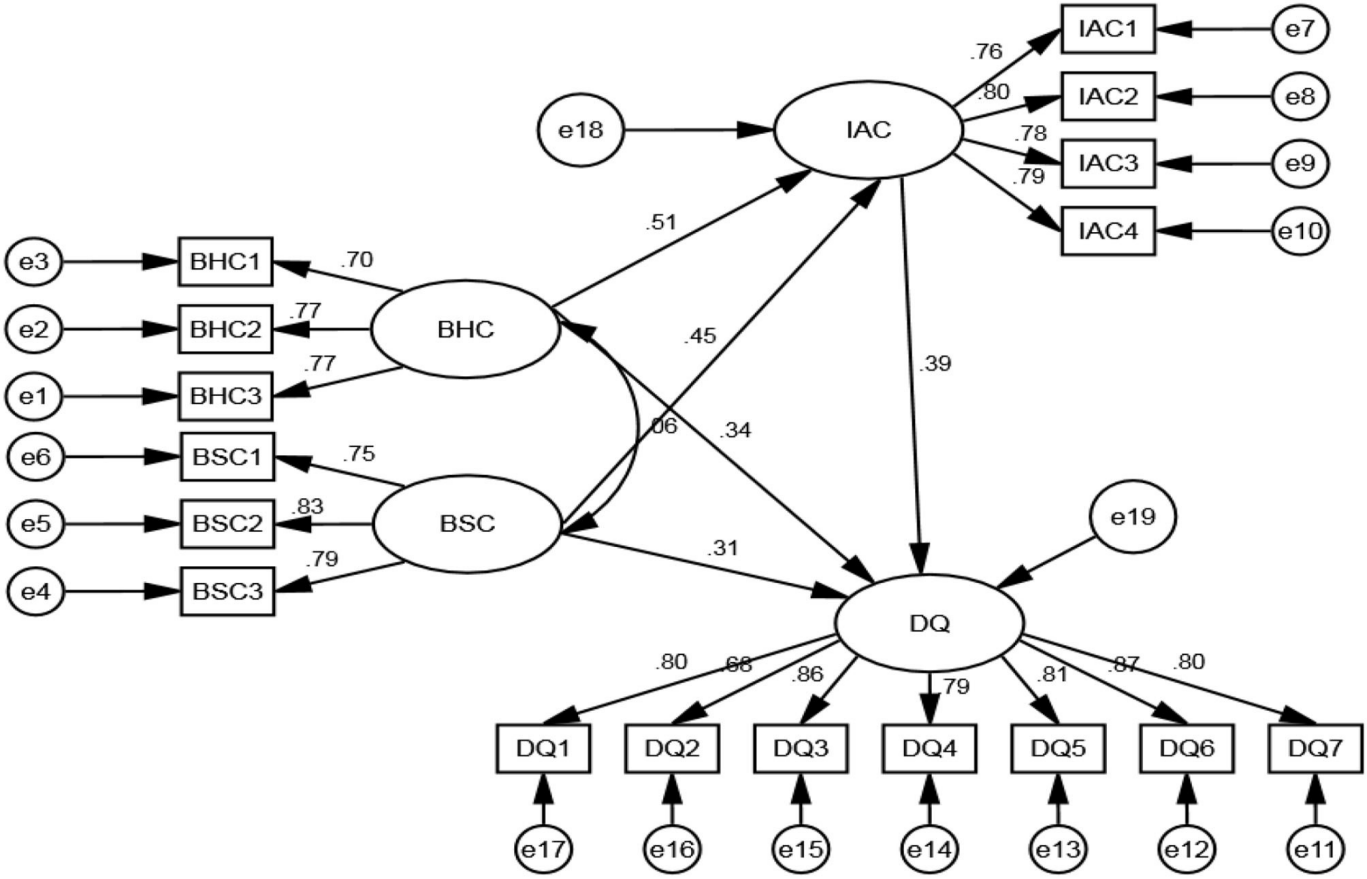
SEM outcomes.

In general, when the degree of freedom is reduced and the model becomes complex (increase the free parameters), the chi-square of the model will be reduced; when the degree of freedom is increased, the model becomes simple (reduce the free parameters), the chi-square of the model will be increased. If the chi-square decreases significantly with the increase of free parameters, it shows that the increase of free parameters is worthwhile (Sekaran and Bougie, 2003). If the chi-square does not increase significantly after the free parameters are reduced, it is advisable to reduce the free parameters. Comparing model M1 with model m2, when ΔDF = 2, Δχ^2^ (1) = 0.52, check if = 2, α = 0.05, the χ^2^ critical value is 5.99, obviously 0.52 is <5.991, so model M1 is better than model m2. Then compare model M3 with model m2, ΔDF = 1, Δχ^2^ (1) = 2.12, check if = 1, α = 0.05, the χ^2^ critical value is 3.84, obviously 2.12 is <3.84, so model M2 is better than model m3. Comparing model M4 with model M5, ΔDF = 1, Δχ^2^ (1) = 1.18, check if = 1, α = 0.05, the χ^2^ critical value is 3.84, obviously 1.88 is <3.84, so model M5 is better than model M4. Continue to compare model M2 with model M5, ΔDF = 1, Δχ^2^ (1) = 5.28, check the χ^2^ critical value when DF = 1, α = 0.05 is 3.84, obviously 5.28 is >3.84, so model M5 is better than model m2. Comparing M1 with model M5, ΔDF = 3, Δχ^2^ (1) = 5.80, check if = 3, α = 0.05, the χ^2^ critical value is 7.82, 5.80 is <7.82, so model M1 is better than model M5. Therefore, M1 is determined as the optimal model (Figure 2 is the path coefficient of M1). As the model M1 is a partial intermediary model, it can be confirmed that the board capital has a direct impact on the green innovation strategy DQ, and it also proves that the green innovation strategic IAC plays a partial intermediary role in the relationship between the two dimensions of board capital and the green innovation strategy DQ.

Further, analyze the path coefficient of M1 (see Figure 2). From the perspective of direct effect, the two dimensions of board capital have a direct positive impact on the quality of green innovation strategic decision-making (β = 0.34, *p* < 0.001, β = 0.31, *p* < 0.001). H1a and H1b are verified. The direct positive effects of the two dimensions of board capital on the green innovation strategic IAC (β = 0.51, *p* < 0.001, β = 0.45, *p* < 0.001), H2a and H2b, are verified. The direct positive effect of the green innovation strategic IAC on the green innovation strategy DQ (β = 0.39, *p* < 0.001), H3, is verified. From the perspective of indirect effect, the two dimensions of board capital have an indirect positive impact on the green innovation strategy DQ through the green innovation strategic IAC, with the effect values of 0.20 (0.51 × 0.39) and 0.16 (0.45 × 0.39), respectively. H4a and H4b are verified. From the perspective of the total effect, the total effect value of BHC on the green innovation strategy DQ is 0.54 (0.34 + 0.20), in which the contribution rate of direct effect is 63%, the contribution rate of indirect effect is 37%; the total effect value of BSC on the green innovation strategy DQ is 0.47 (0.31 + 0.16), in which the contribution rate of direct effect is 66%, and the contribution rate of indirect effect is 34%.

According to the path analysis of M1, it is proved that the green innovation strategic IAC has some intermediary effect on the relationship between the two dimensions of board capital and the green innovation strategy DQ. According to the method proposed by Baron and Kenny (1986), the green innovation strategic IAC plays a part of intermediary role in the relationship between board capital and the green innovation strategy DQ, which must meet four conditions: (1) The two dimensions of board capital (independent variable) are significantly related to the green innovation strategic IAC (intermediary variable); (2) the two dimensions of board capital are significantly related to the green innovation strategy DQ (dependent variable); (3) the green innovation strategic IAC is significantly related to the green innovation strategy DQ; (4) when the green innovation strategic IAC enters into the relationship between the two dimensions of board capital and the green innovation strategy DQ, the relationship between BHC and BSC and the green innovation strategy DQ is significantly weakened. According to Table 4, there is a significant correlation between the independent variable and dependent variable, independent variable and intermediate variable, and intermediate variable and dependent variable. In M5, the two dimensions of board capital have a significant relationship with the green innovation strategy DQ (β = 0.54, *p* < 0.005; β = 0.49, *p* < 0.005, *p* < 0.005). However, after the introduction of the green innovation strategic IAC as the intermediary role, M1 shows that the significant weakening path coefficients of the two dimensions of board capital and the green innovation strategy DQ are reduced to 0.34 (*p* < 0.001) and 0.31 (*p* < 0.001), respectively. H4a and H4b are further verified. The overall results of our study are consistent with the literature (Thatcher and Patel, 2012; De Maere et al., 2014; Makaryanawati, 2019; Ramón-Llorens et al., 2019; Xia et al., 2022).

### Test of Moderated Mediating Effect

In this study, we use the coefficient product method proposed by Hair et al. (2014) to test the moderated mediating effect, that is, to judge whether the moderated mediating effect is significant by testing the significance of the path coefficient product between the interaction term and the mediating variable. At the same time, according to the difference analysis method proposed by Edwards and Lambert (2007), further verification is carried out, that is to say, by directly testing the significance of the difference between the mediating effect under the condition of a strong Faultline and weak BGF, to judge whether the mediating effect with regulation is significant, to test the moderating effect of the intermediary role of the BGF on the ability to obtain innovation strategic information. Using the PROCESS macro written by Hayes running directly in SPSS 21.0, the results are as follows: in the intermediary of the board, human capital, and BSC from the green innovation strategic IAC to the green innovation strategy DQ, the product of path coefficient between interaction item and intermediary variable is −0.023 (*p* < 0.005) and −0.035 (*p* < 0.005), respectively, indicating that the mediating effect is negatively regulated by the BGF.

Using AMOS 24.0 to perform the Bootstrap test on the mediating effect with regulation, and the test results are shown in Table 5. When the degree of BGF is high (a standard deviation above the mean), the mediating effect value of BHC and BSC is 0.13 (*p* < 0.005), 0.09 (*p* < 0.001), and the 95% deviation correction Bootstrap confidence interval is [0.089, 0.303], [0.112, 0.339], excluding 0, and the mediating effect is significant. When the degree of the BGF is low (a standard deviation under the mean), the mediating effect value of BHC and BSC from the green innovation strategic IAC to the green innovation strategy DQ is 0.29 (*p* < 0.005), and the 95% deviation correction Bootstrap confidence interval of 0.20 (*p* < 0.005) is [0.183, 0.551], [0.171, 0.473], excluding 0, and the mediating effect is significant. There is a significant difference between the indirect effect value of the intermediate path when the BGF is high and the indirect effect value of the intermediate path when the BGF is low (*p* < 0.005, CI [−0.094, −0.248]) (*p* < 0.005, CI [−0.059, −0.134]). It shows that the mediating effect of the green innovation strategic IAC between BHC and BSC and the green innovation strategy DQ is significantly enhanced when the degree of the BGF decreases. H5a and H5b are verified.

**Table 5:** Moderated mediating effect.

		**Path 1: BHC-IAC-DQ**			**Path 2: BSC-IAC-DQ**	
**Moderator**	**Indirect effect**	**95%confidence level**		**Indirect effect**	**95% confidence level**	
		**Lower bounds**	**Upper bounds**		**Lower bounds**	**Upper bounds**
High group Faultline	0.132**	0.089	0.303	0.098**	0.112	0.339
Low group Faultline	0.293**	0.183	0.551	0.201**	0.171	0.473
Difference	−0.161**	−0.094	−0.248	−0.103**	−0.059	−0.134

**p <0.005.

## Conclusion, Implication, and Future Directions

### Conclusion

This study divides the technology green strategic IAC and the BGF into two parts: the intermediary variable and the regulating variable. It discusses the mechanism of the two dimensions of board capital on the green innovation strategy DQ and makes an empirical analysis with 230 enterprises as research samples. The results show that: (1) The two dimensions of board capital have a significant positive impact on the green innovation strategy DQ. (2) The green innovation strategic IAC plays the part of a mediator in the association between the board capital and the green innovation strategy DQ. (3) The BGF plays a negative moderating role in the intermediary role of the green innovation strategic IAC in the association between the board capital and the green innovation strategy DQ.

### Theoretical Contribution

The theoretical contributions of this study are as follows: (1) It enriches and strengthens the evidence that board capital is a positive factor in the green innovation strategy DQ. In the past, most scholars studied the impact of board capital on the green innovation strategy DQ theoretically, but empirical research is still relatively small, and there is no consistent conclusion. Through empirical analysis, this study shows that the two dimensions of board capital have a significant positive impact on the green innovation strategy DQ, which supports the views of Westphal and Bednar (2005) and Liu et al. (2010). In the provision of new evidence for the positive impact of board capital on the green innovation strategy DQ the influence path of board capital on the green innovation strategy DQ is explored. From the perspective of acquisition of innovation strategic information, this study constructs the function routes of “BHC—the green innovation strategic IAC—the green innovation strategy DQ” and “BSC—the green innovation strategic IAC—the green innovation strategy DQ” and opens the “black box” between them. (3) It expands the boundary conditions of board capital research. In this study, BGF is incorporated into the research framework to investigate the mediating role of technological innovation strategic information acquisition ability on the relationship between board capital and green innovation strategic decision quality under different degrees of the board faultline. It deepens the boundary conditions of the board capital's effect on the quality of green innovation strategic decisions. It also explains the divergence of the research on the relationship between board capital and the quality of green innovation strategic decision to a certain extent. This opens a new direction for researchers to explore the literary development of this concept.

### Management Implications

The management implications of this study are (1) The board should fully accumulate human capital and social capital. The board members should actively participate in training and continuous learning to improve BHC inventory and quality. Similarly, they should take the initiative to strengthen social communication with external organizations, including not only traditional customers, suppliers, or partners, but also government departments, scientific research institutions, consulting institutions, financial institutions, etc. It will help to provide important support for the strategic decision of high-quality innovation. (2) The board should fully recognize the key role of the technology innovation strategic IAC. The board members should summarize the practical experience, strengthen information awareness, cultivate the ability to change the information demand of innovation strategy, select and use innovation strategy information. So that the capital of the board of directors can be continuously transformed into the improvement of the quality of innovation strategy decisions. (3) Enhance the sense of belonging of each director and reduce the degree of BGF. Through unstructured meetings and other forms, increase the opportunities for informal interaction of the board of directors, promote the sense of belonging of board members to the whole company, increase the cohesion of the board team, and weaken the negative impact of BGF.

### Limitations and Future Directions

Although this research has contributions, there are still some constraints. First, the data cross-section design makes the research results have some common method bias. Although the confirmatory factor analysis has been passed, the impact cannot be completely excluded. In the future, longitudinal design can be used for further verification. Second, this paper finds that the green innovation strategic IAC acts as a part of intermediary, which shows that the relationship between board capital and the green innovation strategy DQ is quite complex, and there may be other influencing factors, such as the cognitive ability of the board and the strategic decision-making ability of innovation. In the future, we can continue to explore other relationship mechanisms from the above perspectives.

## Data Availability Statement

The raw data supporting the conclusions of this article will be made available by the authors, without undue reservation.

## Ethics Statement

The studies involving human participants were reviewed and approved by School of Economics and Management, Harbin University. The Ethics Committee waived the requirement of written informed consent for participation.

## Author Contributions

JF: conceptualization, writing—original draft preparation, data curation, software, and formal analysis. YP: supervision, methodology, fund acquisition, and project administration. WZ: data curation, visualization, writing—review and editing, and validation. All authors contributed to the article and approved the submitted version.

## Funding

Support from Heilongjiang Province Philosophy and Social Science Research and Planning Project (Project No. 18JYB152) is gratefully acknowledged.

## Conflict of Interest

The authors declare that the research was conducted in the absence of any commercial or financial relationships that could be construed as a potential conflict of interest.

## Publisher's Note

All claims expressed in this article are solely those of the authors and do not necessarily represent those of their affiliated organizations, or those of the publisher, the editors and the reviewers. Any product that may be evaluated in this article, or claim that may be made by its manufacturer, is not guaranteed or endorsed by the publisher.

## References

[B1] Ali M. Ayoko O. B. (2020). The impact of board size on board demographic faultlines. Corp. Gov. Int. J. Bus. Soc. 20, 1205–1222. 10.1108/CG-03-2020-0100

[B2] Azevedo L (2021). Board capital and board effectiveness: an examination of Florida community foundations. J. Nonprofit Educ. Leadersh. 12, 52–71. 10.18666/JNEL-2021-10775

[B3] Barney J. B (2001). Is the resource-based “view” a useful perspective for strategic management research? Yes. Acad. Manag. Rev. 26, 41–56. 10.5465/AMR.2001.4011938

[B4] Baron R. M. Kenny D. A. (1986). The moderator-mediator variable distinction in social psychological research. Conceptual, strategic, and statistical considerations. J. Pers. Soc. Psychol. 51, 1173–1182. 10.1037/0022-3514.51.6.1173 3806354

[B5] Benson V. Ezingeard J. N. Hand C. (2019). An empirical study of purchase behaviour on social platforms: the role of risk, beliefs and characteristics. Inf. Technol. People 32, 876–896. 10.1108/ITP-08-2017-0267

[B6] Bezrukova K. Thatcher S. M. B. Jehn K. A. (2007). “Group heterogeneity and faultlines: comparing alignment and dispersion theories of group composition,” in Conflict in Organizational Groups: New Directions in Theory and Practice, eds. L. Thompson and K. J. Behfar (Evanston, IL: Northwestern University Press), 57–92. Available online at: https://nupress.northwestern.edu/9780810124578/conflict-in-organizational-groups/

[B7] Bezrukova K. Thatcher S. M. B. Jehn K. A. Spell C. S. (2012). The effects of alignments: examining group faultlines, organizational cultures, and performance. J. Appl. Psychol. 97, 77–92. 10.1037/a0023684 21744943

[B8] Byrd T. A. Turner D. E. (2000). Measuring the flexibility of information technology infrastructure: Exploratory analysis of a construct. J. Manag. Inf. Syst. 17, 167–208. 10.1080/07421222.2000.11045632

[B9] Chen H. L (2014). Board capital, CEO power and RandD investment in electronics firms. Corp. Gov. An Int. Rev. 22, 422–436. 10.1111/corg.12076

[B10] Crucke S. Knockaert M. (2016). When stakeholder representation leads to faultlines: a study of board service performance in social enterprises. J. Manag. Stud. 53, 768–793. 10.1111/joms.12197

[B11] Dakhli M. De Clercq D. (2004). Human capital, social capital, and innovation: a multi-country study. Entrep. Reg. Dev. 16, 107–128. 10.1080/08985620410001677835

[B12] De Maere J. Jorissen A. Uhlaner L. M. (2014). Board capital and the downward spiral: antecedents of bankruptcy in a sample of unlisted firms. Corp. Gov. Int. Rev. 22, 387–407. 10.1111/corg.12078

[B13] Dooley R. S. Fryxell G. E. (1999). Attaining decision quality and commitment from dissent: the moderating effects of loyalty and competence in strategic decision-making teams. Acad. Manag. J. 42, 389–402. 10.2307/257010

[B14] Edwards J. R. Lambert L. S. (2007). Methods for integrating moderation and mediation: A general analytical framework using moderated path analysis. Psychol. Methods 12, 1–22. 10.1037/1082-989X.12.1.1 17402809

[B15] Ewer M. S. Carver J. R. Minotti G. (2019). Old and new directions of Cardio-Oncology. Semin. Oncol. 46, 395–396. 10.1053/j.seminoncol.2019.11.003 31767271

[B16] Fischer H. M. Pollock T. G. (2004). Effects of social capital and power on surviving transformational change: the case of initial public offerings. Acad. Manag. J. 47, 463–481. 10.5465/20159597

[B17] Forbes D. P (2007). Reconsidering the strategic implications of decision comprehensiveness. Acad. Manag. Rev. 32, 361–376. 10.5465/AMR.2007.24349585

[B18] Gagné M. Deci E. L. (2005). Self-determination theory and work motivation. J. Organ. Behav. 26, 331–362. 10.1002/job.322

[B19] González-Valiente C. L. Santos M. L. Arencibia-Jorge R. (2019). Evolution of the socio-cognitive structure of knowledge management (1986–2015): an author co-citation analysis. J. Data Inf. Sci. 4, 36–55. 10.2478/jdis-2019-0008

[B20] Haas L. Jordan S. C. Wisheart J. (1981). Fulminant *Streptococcus pyogenes* infection. Br. Med. J. 282, 399. 10.1136/bmj.282.6261.399-a 6780039 PMC1504158

[B21] Hair J. F. Sarstedt M. Hopkins L. Kuppelwieser V. G. (2014). Partial least squares structural equation modeling (PLS-SEM): an emerging tool in business research. Eur. Bus. Rev. 26, 106–121. 10.1108/EBR-10-2013-0128

[B22] Hillman A. J. Dalziel T. (2003). Boards of directors and firm performance: Integrating agency and resource dependence perspectives. Acad. Manag. Rev. 28, 383–396. 10.5465/AMR.2003.10196729

[B23] Hu R. Shahzad F. Abbas A. Liu X. (2022). Decoupling the influence of eco-sustainability motivations in the adoption of the green industrial IoT and the impact of advanced manufacturing technologies. J. Clean. Prod. 339, 130708. 10.1016/j.jclepro.2022.130708

[B24] Jermias J. Gani L. (2014). The impact of board capital and board characteristics on firm performance. Br. Account. Rev. 46, 135–153. 10.1016/j.bar.2013.12.001

[B25] Johnson J. L. Daily C. M. Ellstrand A. E. (1996). Boards of directors: a review and research agenda. J. Manage. 22, 409–438. 10.1016/S0149-2063(96)90031-8

[B26] Karimi Takalo S. Sayyadi Tooranloo H. Shahabaldini parizi Z. (2021). Green innovation: a systematic literature review. J. Clean. Prod. 279, 122474. 10.1016/j.jclepro.2020.122474

[B27] Kontesa M. Lako A. Wendy W. (2020). Board capital and earnings quality with different controlling shareholders. Account. Res. J. 33, 593–613. 10.1108/ARJ-01-2020-0017

[B28] Li X. Du J. Long H. (2019). Theoretical framework and formation mechanism of the green development system model in China. Environ. Dev. 32, 100465. 10.1016/j.envdev.2019.100465

[B29] Lim J. Y. K. Busenitz L. W. Chidambaram L. (2013). New venture teams and the quality of business opportunities identified: faultlines between subgroups of founders and investors. Entrep. Theory Pract. 37, 47–67. 10.1111/j.1540-6520.2012.00550.x

[B30] Lin C. Y. Y. Wei Y. C. Chen M. H. (2006). The role of board chair in the relationship between board human capital and firm performance. Int. J. Bus. Gov. Ethics 2, 329–340. 10.1504/IJBGE.2006.011161

[B31] Liu D. Liang Y. Zhang L. Zhang Y. (2010). “The effects of human capital on competitive strategies and performance – evidence from listed companies in China's SME board,” in 5th IEEE International Conference on Management of Innovation and Technology, ICMIT2010, 670–675. 10.1109/ICMIT.2010.5492740

[B32] Makaryanawati (2019). “The effect of human capital on the role of the board of commissioners,” in Proceedings of the 3rd International Conference on Accounting, Management and Economics 2018 (ICAME 2018) (Paris: Atlantis Press). 10.2991/icame-18.2019.23

[B33] Malik B. H. Shuqin C. Mastoi A. G. Ahmed Ghais A. H. A. (2016). Citizen's adoption of mobile land record information systems (mLRMIS): a case of Pakistan. Eur. Sci. Journal, ESJ 12, 393. 10.19044/esj.2016.v12n5p393

[B34] Meissner P. Wulf T. (2013). Cognitive benefits of scenario planning: its impact on biases and decision quality. Technol. Forecast. Soc. Change 80, 801–814. 10.1016/j.techfore.2012.09.011

[B35] Müller J. M. Buliga O. Voigt K.-I. (2021). The role of absorptive capacity and innovation strategy in the design of industry 4.0 business models – a comparison between SMEs and large enterprises. Eur. Manag. J. 39, 333–343. 10.1016/j.emj.2020.01.002

[B36] Myon E. Martin N. Taieb C. (2005). The French aging males' symptoms (AMS) scale: methodological review. Health Qual. Life Outcomes 3, 20. 10.1186/1477-7525-3-20 15790411 PMC1079916

[B37] Nahl D. Tenopir C. (1996). Affective and cognitive searching behavior of novice end-users of a full-text database. J. Am. Soc. Inf. Sci. 47, 276–286. 10.1002/(SICI)1097-4571(199604)47:4<276::AID-ASI3>3.0.CO;2-U

[B38] Nutt P. C (2008). Investigating the success of decision making processes. J. Manag. Stud. 45, 425–455. 10.1111/j.1467-6486.2007.00756.x

[B39] O'Brien H. L. Symons S. (2005). The information behaviors and preferences of undergraduate students. Res. Strateg. 20, 409–423. 10.1016/j.resstr.2006.12.02131140943

[B40] Park E. Kim K. J. (2014). An integrated adoption model of mobile cloud services: exploration of key determinants and extension of technology acceptance model. Telemat. Inf. 31, 376–385. 10.1016/j.tele.2013.11.008

[B41] Rafferty A. E. Gary M. S. (2016). Enhancing the success of mergers and acquisitions: a theory-driven approach. Acad. Manag. Proc. 2016, 10294. 10.5465/ambpp.2016.10294abstract

[B42] Ramón-Llorens M. C. García-Meca E. Pucheta-Martínez M. C. (2019). The role of human and social board capital in driving CSR reporting. Long Range Plann. 52, 101846. 10.1016/j.lrp.2018.08.001

[B43] Rui-Zhi Y. Wei G. Hui-Hui J. (2019). Influence of board capital on enterprise innovation performance in uncertain environment, in a dynamic-capability perspective. J. Environ. Prot. Ecol. 20, 924–933.

[B44] Sarto F. Saggese S. Viganò R. Mauro M. (2019). Human capital and innovation: mixing apples and oranges on the board of high-tech firms. Manag. Decis. 58, 897–926. 10.1108/MD-06-2017-0594

[B45] Schiederig T. Tietze F. Herstatt C. (2012). Green innovation in technology and innovation management – an exploratory literature review. R & D Manag. 42, 180–192. 10.1111/j.1467-9310.2011.00672.x

[B46] Sekaran U. Bougie R. (2003). Research Method for Business: A Skill Building Approach, 5th Edn. Illinois, IL: United States John Wiley Sons Inc., 23.

[B47] Shahzad F. Abbas A. Fateh A. Kasim R. S. R. Akram K. Ashraf S. F. (2021). Late-night use of social media and cognitive engagement of female entrepreneurs: a stressor–strain–outcome perspective. SAGE Open 11, 1–21. 10.1177/21582440211037652

[B48] Shahzad F. Du J. Khan I. Wang J. (2022). Decoupling institutional pressure on green supply chain management efforts to boost organizational performance: moderating impact of big data analytics capabilities. Front. Environ. Sci. 10:911392. 10.3389/fenvs.2022.911392

[B49] Shin T. You J. (2019). The effects of board faultlines on CEO dismissal. Acad. Manag. Proc. 2019, 16697. 10.5465/AMBPP.2019.16697abstract

[B50] Song W. Yu H. (2018). Green innovation strategy and green innovation: the roles of green creativity and green organizational identity. Corp. Soc. Responsib. Environ. Manag. 25, 135–150. 10.1002/csr.1445

[B51] Thatcher S. M. B. Jehn K. A. Zanutto E. (2003). Cracks in diversity research: the effects of diversity faultlines on conflict and performance. Gr. Decis. Negot. 12, 217–241. 10.1023/A:1023325406946

[B52] Thatcher S. M. B. Patel P. C. (2012). Group faultlines: a review, integration, and guide to future research. J. Manage. 38, 969–1009. 10.1177/0149206311426187

[B53] Thomas G. F. Zolin R. Hartman J. L. (2009). The central role of communication in developing trust and its effect on employee involvement. J. Bus. Commun. 46, 287–310. 10.1177/0021943609333522

[B54] Thong J. Y. L. Yap C. S. Raman K. S. (1996). Top management support, external expertise and information systems implementation in small businesses. Inf. Syst. Res. 7, 248–267. 10.1287/isre.7.2.248

[B55] Trezzini B (2008). Probing the group faultline concept: an evaluation of measures of patterned multi-dimensional group diversity. Qual. Quant. 42, 339–368. 10.1007/s11135-006-9049-z

[B56] Valenti A. Horner S. (2020). The human capital of boards of directors and innovation: an emppirical examination of the pharmaceutical industry. Int. J. Innov. Manag. 24, 2050056. 10.1142/S1363919620500565

[B57] Wakolbinger T. Cruz J. M. (2011). Supply chain disruption risk management through strategic information acquisition and sharing and risk-sharing contracts. Int. J. Prod. Res. 49, 4063–4084. 10.1080/00207543.2010.501550

[B58] Wang M. Y. Zhang P. Z. Zhou C. Y. Lai N. Y. (2019). Effect of emotion, expectation, and privacy on purchase intention in wechat health product consumption: the mediating role of trust. Int. J. Environ. Res. Public Health 16, 3861. 10.3390/ijerph16203861 31614749 PMC6843468

[B59] Wang R. T. Lin C. P. (2012). Understanding innovation performance and its antecedents: a socio-cognitive model. J. Eng. Technol. Manag. 29, 210–225. 10.1016/j.jengtecman.2012.01.001

[B60] Wang T (2021). Board human capital diversity and corporate innovation: a longitudinal study. Corp. Gov. Int. J. Bus. Soc. 22, 680–701. 10.1108/CG-03-2021-0126

[B61] Westphal J. D. Bednar M. K. (2005). Pluralistic ignorance in corporate boards and firms' strategic persistence in response to low firm performance. Adm. Sci. Q. 50, 262–298. 10.2189/asqu.2005.50.2.262

[B62] Wilson T. D (1997). Information behaviour: an interdisciplinary perspective. Inf. Process. Manag. 33, 551–572. 10.1016/S0306-4573(97)00028-9

[B63] Wilson T. D (1999). Models in information behaviour research. J. Doc. 55, 249–270. 10.1108/EUM0000000007145

[B64] Wincent J. Anokhin S. Örtqvist D. (2010). Does network board capital matter? A study of innovative performance in strategic SME networks. J. Bus. Res. 63, 265–275. 10.1016/j.jbusres.2009.03.012

[B65] Wood R. Bandura A. (1989). Social cognitive theory of organizational management. Acad. Manag. Rev. 14, 361–384. 10.5465/amr.1989.4279067

[B66] Xia L. Gao S. Wei J. Ding Q. (2022). Government subsidy and corporate green innovation – does board governance play a role? Energy Policy 161, 112720. 10.1016/j.enpol.2021.112720

[B67] Yang H. Lin Z. Lin Y. (2010). A multilevel framework of firm boundaries: firm characteristics, dyadic differences, and network attributes. Strateg. Manag. J. 31, 237–261. 10.1002/smj.815

[B68] Zhang J. Liang G. Feng T. Yuan C. Jiang W. (2020a). Green innovation to respond to environmental regulation: how external knowledge adoption and green absorptive capacity matter? Bus. Strateg. Environ. 29, 39–53. 10.1002/bse.2349

[B69] Zhang Y. Ma L. (2022). Board faultlines, innovation strategy decisions, and faultline activation: research on technology-intensive enterprises in Chinese A-share companies. Front. Psychol. 13:855610. 10.3389/fpsyg.2022.855610 35432067 PMC9009146

[B70] Zhang Y. Sun J. Yang Z. Wang Y. (2020b). Critical success factors of green innovation: technology, organization and environment readiness. J. Clean. Prod. 264, 121701. 10.1016/j.jclepro.2020.121701

[B71] Zhang Z (2019). Visualization analysis of the development trajectory of knowledge sharing in virtual communities based on CiteSpace. Multimed. Tools Appl. 78, 29643–29657. 10.1007/s11042-018-6061-y

